# A Social Reform Club

**Published:** 1895-11-09

**Authors:** 


### Modern Sociology.

### A SOCIAL REFORM CLUB.

In New York there has been formed a "Social Reform Club" with a view to improving the relations between employers and employed. If this club fulfils the object for which it is established it will do a most useful work, for, bitter as are the feelings with which masters and workmen regard each other here, they are "as moonlight unto sunlight," and as water unto—vinegar, compared with the class hatreds of the United States. And certainly the objects of the club, as stated in its prospectus, commend themselves both by what its members hope to do and what they purpose to leave undone. The first object is: "To form a common centre at which wage earners and others interested in the labour movement may meet to consider the next step or steps which should be taken in order to improve industrial and social conditions in the city of New York." So far, good; but to us who have known so many societies with vague Utopian aims which thought to do so much and did so little, this large ambition seems to be most wisely limited and conditioned by the second object, "The specific characteristic of this club is to be that it shall take no share in the propaganda of general theories of society, and shall rigidly exclude all so-called 'social panaceas' from its discussions, but shall confine itself to the consideration, the advocacy, and the carrying out of practical measures such as can be undertaken in the immediate future with fair hope of success, and commend themselves to conscience and to common sense."

This is good; this is worthy of a nation which is above all direct, practical, and clear-sighted; which in their own idiom, "gets there." They have got there—to the heart of the social problem, when they discard in advance the "social panacea." We hear so much of schemes of social reform which, translated into practical language, begin thus: First get rid of original sin. Make all employers just and considerate, all workmen honest and industrious, and then—why, then no schemes, no reforms will be needed. The New York Social Reform Club has perceived this, and despairing of eliminating human nature with all its faults from the labour world, confines its attention to "measures such as can be undertaken in the immediate future with fair hope of success."

The constitution of the club is framed so as to do justice to both parties. The membership is to consist as far as possible in equal proportions of wage-workers and of those who are interested in social reform, and the executive council is to consist of seven members who are wage-earners, seven who are not, and a chairman of either class whom these elect. That is to say, the balance of power, to use a phrase more European than American, shall be maintained. Five standing committees are to be formed from the members, dealing with, in the first place, the admission of new members, but taking up wider subjects. One, the Committee on Legislation, is to prepare and, with the approval of the club, present to the legislative bodies of the State Bills which tend to promote the welfare of the wage-workers. Another committee will aid in protecting the rights and interests of the wage-earner in Court, whenever general questions are raised affecting those rights and interests. Perhaps there is no country where such an advisory committee is not needed, and certainly in the United States the Courts are too corrupt to render it unnecessary. Another, but kindred committee, is to insist on the enforcement of laws affecting social reform. The last committee has a wider task. This is the "Committee of Instruction." Three duties are laid upon it; first, the enlightenment of the public on the necessity of organised relations between employers and employés; second, the inculcation of a spirit of friendly co-operation between the members of all classes in the solution of the labour problem; third, the public discussion and the advocacy, on platforms and in print, of such special measures as the club may from time to time agree upon. The club means to exert its influence on public opinion through the press and otherwise, especially in the case of strikes. When either strikes or lock-outs occur the executive council of the club is to do its best to promote conciliation and arbitration. If these methods fail, it purposes to investigate the merits of the question at issue, and then use its influence and its money on behalf of whichever side justice may be found.

If such a tribunal keeps itself really balanced and impartial its value may be immense. It will, of course, be laughed at to begin with; but if it once proves itself just and impartial, both sides will in the end seek its arbitration. But it will require to consist of the right men. We on this side know of whom such associations are apt to consist. Workmen who cannot work, employers who cannot guide, meet and dream over the world as it can never be. If the employers come to bankruptcy and the workmen to the poorhouse through indulgence in impossible theories, another set arise to take their place. It is useless for wage-earners to belong to such a club who are not willing to admit that their master gives thought and capital to his enterprise, and is entitled to some recompense for these, even if he never "takes his coat off his back." It is useless for employers to join it who cannot see that the man who does their bidding should profit by any improvement of prices, any enlargement of market, as well as he. And it is useless for the club to attempt any of its functions if once it gets the reputation of favouring one side, even the side against which the world, the flesh, and the devil are most generally arrayed. Justice for the rich is needed as well as for the poor, and in order that the poor may have justice, and may not be prejudiced in the world's eyes there must be no one-sidedness by their champions. But if conducted as it hopes to be with conscience and commonsense the club may do work of endless value in the labour world.

The question arises, are not such clubs possible and desirable with us? We, too, have labour disputes; we are in the midst of one just now. Is it not possible to create some tribunal where both parties would be represented, not by the irreconcileables of each, but by men who can look with impartial eyes on the rights and wrongs of either side? And apart from direct arbitration, would not a more wholesome frame of mind arise from the existence in our midst of a club which represented both parties, and could see that the interests in which masters and men are united are greater than those in which they are opposed?

